# Impaired Spermatogenesis in Infertile Patients with Orchitis and Experimental Autoimmune Orchitis in Rats

**DOI:** 10.3390/biology13040278

**Published:** 2024-04-19

**Authors:** María Sofía Amarilla, Leilane Glienke, Thaisy Munduruca Pires, Cristian Marcelo Sobarzo, Hernán Gustavo Oxilia, María Florencia Fulco, Marcelo Rodríguez Peña, María Belén Maio, Denisse Ferrer Viñals, Livia Lustig, Patricia Verónica Jacobo, María Susana Theas

**Affiliations:** 1 Instituto de Investigaciones Biomédicas (INBIOMED), CONICET-Universidad de Buenos Aires, Paraguay 2155, Piso 10, Laboratorio 10, Ciudad Autónoma de Buenos Aires C1421ABG, Argentina; glienke@campus.fmed.uba.ar (L.G.); munduruca.thaisy@campus.fmed.uba.ar (T.M.P.); csobarzo@fmed.uba.ar (C.M.S.); mbelenmaio@campus.fmed.uba.ar (M.B.M.); dfvinals1456@gmail.com (D.F.V.); llustig@fmed.uba.ar (L.L.);; 2 Departamento de Biología Celular, Facultad de Medicina, Universidad de Buenos Aires, Cátedra II de Histología, Paraguay 2155, Ciudad Autónoma de Buenos Aires C1421ABG, Argentina; hoxilia@fmed.uba.ar; 3 Anatomía Patológica, Hospital General de Agudos Parmenio Piñero, Varela 1301, Ciudad Autónoma de Buenos Aires C1406ELA, Argentina; 4 Hospital de Clínicas General San Martín, Av. Córdoba 2351 (C1120AAR), Ciudad Autónoma de Buenos Aires C1421ABG, Argentina; info@doctorafulco.com.ar (M.F.F.); mrodriguez@hmc.mil.ar (M.R.P.)

**Keywords:** experimental orchitis, male infertility spermatogonia, Sertoli cells, spermatogenesis

## Abstract

**Simple Summary:**

In the testis of infertile patients, we have demonstrated a high prevalence of immune cells (orchitis); their presence together with high FSH levels are associated with alterations in the behavior of nurturing Sertoli cells, which are mostly immature, in the disturbed balance of death and proliferation of germ cells facts leading to reduced sperm production. The experimental model of infertility in rats associated with chronic testicular inflammation, experimental autoimmune orchitis (EAO), shares a common hormonal profile and Sertoli cell and germ cell behavior with orchitis in humans. We support EAO as a useful tool to study how infertility is initiated and progresses, and to search for therapies to improve and/or restore gamete production in infertile patients.

**Abstract:**

Experimental autoimmune orchitis (EAO) is a well-established rodent model of organ-specific autoimmunity associated with infertility in which the testis immunohistopathology has been extensively studied. In contrast, analysis of testis biopsies from infertile patients associated with inflammation has been more limited. In this work, testicular biopsies from patients with idiopathic non-obstructive azoospermia diagnosed with hypospermatogenesis (HypoSp) [mild: *n* = 9, and severe: *n* = 11], with obstructive azoospermia and complete Sp (spermatogenesis) (control group, C, *n* = 9), and from Sertoli cell-only syndrome (SCOS, *n* = 9) were analyzed for the presence of immune cells, spermatogonia and Sertoli cell (SCs) alterations, and reproductive hormones levels. These parameters were compared with those obtained in rats with EAO. The presence of increased CD45+ cells in the seminiferous tubules (STs) wall and lumen in severe HypoSp is associated with increased numbers of apoptotic meiotic germ cells and decreased populations of undifferentiated and differentiated spermatogonia. The SCs showed an immature profile with the highest expression of AMH in patients with SCOS and severe HypoSp. In SCOS patients, the amount of SCs/ST and Ki67+ SCs/ST increased and correlated with high serum FSH levels and CD45+ cells. In the severe phase of EAO, immune cell infiltration and apoptosis of meiotic germ cells increased and the number of undifferentiated and differentiated spermatogonia was lowest, as previously reported. Here, we found that orchitis leads to reduced sperm number, viability, and motility. SCs were mature (AMH-) but increased in number, with Ki67+ observed in severely damaged STs and associated with the highest levels of FSH and inflammatory cells. Our findings demonstrate that in a scenario where a chronic inflammatory process is underway, FSH levels, immune cell infiltration, and immature phenotypes of SCs are associated with severe changes in spermatogenesis, leading to azoospermia. Furthermore, AMH and Ki67 expression in SCs is a distinctive marker of severe alterations of STs in human orchitis.

## 1. Introduction

Male infertility is idiopathic in most cases, but in 8.5% of cases, it is associated with infections or inflammatory processes of the male urogenital tract [1]. The high prevalence of urogenital infections among azoospermic men underpins their role as significant etiologic factors in male infertility [2].

A major obstacle to determining how inflammation impairs testicular function in men is that chronic inflammation is asymptomatic in most patients and tissue inflammatory processes usually occur long before the patient visits a physician for fertility evaluation [3]. Furthermore, diagnosis is only possible using invasive methods such as a testis biopsy followed by histopathology.

Experimental models of infertility associated with chronic inflammation, such as experimental autoimmune orchitis (EAO), are relevant as they provide a valuable tool to study how processes are initiated and progress, as suggested by Naito et al. (2012) [4] and Fijak et al. (2018) [5]. In EAO, inflammatory agents (nitric oxide, TNFα, IL6, IFNγ, and others) produced by infiltrating lymphomonocytes alter spermatogenesis, primarily targeting germ cells and Sertoli cells and also disrupting testosterone production via Leydig cells [6].

The aim of this work was a more comprehensive study of idiopathic testicular inflammation in men to provide new tools for its study and understanding. It focused on the impact of immune cells and Sertoli cell phenotypes on the behavior of spermatogonia, sperm production, and the endocrine profile, data that were compared with those found in rats with EAO [3,7,8,9].

## 2. Material and Methods

### 2.1. Infertile Patients

Testicular biopsies were obtained from 38 azoospermic patients undergoing sperm extraction for assisted fertilization procedures at the Hospital de Clínicas General San Martín (Ciudad Autónoma de Buenos Aires; Argentina).

The protocol for obtaining and processing testicular biopsies was approved by the Ethics Committee of the Hospital de Clínicas General San Martín (Protocol Number: 1031-19) and the Hospital de Pediatría Prof. Dr. Juan P. Garrahan (Protocol Number 864), Ciudad Autónoma de Buenos Aires, Argentina. Patients were identified by numbers according to the chronological order in which the testicular biopsies were obtained; their identity was kept confidential according to the International Data Protection Act (25326). Protocols involving human subjects comply with the Declaration of Helsinki. Patients gave their informed written consent.

Inclusion criteria were clinical diagnosis of azoospermia and normal karyotype. Exclusion criteria were patients with a known cause of infertility, e.g., varicocele and cryptorchidism.

Testis from a 7-month-old boy, who died due to congenital heart disease, was collected at necropsy and used as a prepubertal control. This protocol was approved by the Research Committee of the Hospital de Pediatría Prof. Dr. Juan P. Garrahan.

### 2.2. Histopathological Classification of Testicular Biopsies

Patients with obstructive azoospermia and histopathology compatible with complete spermatogenesis (*n* = 9) were considered as the control group, while the experimental group consisted of patients with non-obstructive azoospermia (*n* = 20).

Biopsies from the experimental group were histopathologically classified as mild hypospermatogenesis (*n* = 9) or severe hypospermatogenesis (*n* = 11) according to the criteria of Mc Lachlan et al. (2007) [10] with minor modifications. Biopsies from patients with Sertoli syndrome alone (SCOS) (*n* = 9) were also examined, although this pathology is associated with genetic causes.

Biopsied tissues were fixed in Bouin, embedded in paraffin, and processed for histopathological analysis and immunohistochemical techniques.

The thickness of ST walls was assessed in periodic acid-Schiff (PAS)-stained sections.

### 2.3. Animals

Male inbred Wistar rats were purchased from Bioterio Central, Facultad de Farmacia y Bioquímica (Buenos Aires, Argentina). Animals were housed at 22 °C with a 12 h light, 12 h dark cycle and fed standard food pellets and water ad libitum. The use of rats followed the National Institutes of Health (NIH) guidelines for the care and use of experimental animals, and ethical approval was granted by local the committee: CICUAL Facultad de Medicina, Universidad de Buenos Aires [RES (CD): 2029/2019].

### 2.4. Induction of Experimental Autoimmune Orchitis (EAO)

Adult rats in the experimental group (EAO) were actively immunized (subcutaneously) at 14-day intervals with testicular homogenate (TH) emulsified with complete Freund’s adjuvant (CFA; Sigma-Aldrich, St. Louis, MO, USA) in the hind footpads, different sites of flanks, and near the neck area (subcutaneously) as described previously [9]. The first two immunizations were followed by an intravenous injection of 10^10^ inactivated *Bordetella pertussis* bacteria (strain 10536; kindly provided by Instituto Malbrán, Buenos Aires, Argentina), whereas the third was followed by an intraperitoneal injection of 5 × 10^9^ *B. pertussis*. Non-immunized adult untreated rats were used as a control group. Rats from the EAO group were killed 50–60 (focal EAO) and 70–80 days (severe EAO) after the first immunization together with rats from the control group. Testes were removed, weighed, fixed in Bouin’s solution or PFA 4%, and embedded in paraffin. Trunk blood samples were taken to measure serum prolactin by RIA.

### 2.5. CD45 Immunohistochemistry

Testis sections from human biopsies were deparaffinized and hydrated by successive series of ethanol and then irradiated in a microwave oven (370 W for 15 min) in 0.01 mM sodium citrate buffer, pH 6.0; then endogenous peroxidase activity was blocked by treatment with 0.03% H_2_O_2_ in methanol absolute (Merck, Baltimore, MD, USA) for 30 min at room temperature (RT). Nonspecific binding sites were blocked with 5% normal horse serum (NHS, Vector Labs, Burlingame, CA, USA) and 20% avidin (Blocking Kit, Vector Labs) in phosphate-buffered saline (PBS) for 30 min at RT. Immune cells were determined using commercially available Monoclonal Mouse Anti-Human CD45, Leucocyte Common Antigen (1:300, 0.375 mg/mL, DAKO, Santa Barbara, CA, USA) for 24 h at 4 °C followed by a horse biotinylated anti-mouse secondary antibody (1/100, 1.5 mg/mL, Vector Labs) in PBS containing 5% NHS for 1 h at RT. The avidin–biotin system (Vectastain Elite ABC kit; Vector Laboratories, Burlingame, CA, USA) was applied for 45 min at RT and the reaction product was visualized by adding diaminobenzidine substrate (Vector Laboratories). In the negative control, the first antibody was omitted. Sections were counterstained with hematoxylin, dehydrated by successive series of ethanol, and mounted.

The number of CD45-immunoreactive cells was quantified in 6 non-consecutive cross-sections using a Nikon microscope at 40× magnification. The area of each section was determined using the same microscope at 2.5× magnification and Image J software 1.52a. Results were expressed as immunoreactive CD45 cells/mm^2^.

### 2.6. Ki67 Immunohistochemistry

The Bouin-fixed paraffin-embedded biopsies and PFA-fixed paraffin-embedded rat testes were subjected to immunohistochemical analysis using the VENTANA Roche automated immunostainer (Roche, Mannheim, Germany). The VENTANA primary Ki67 antibody (Roche) has been developed for use on BenchMark IHC/ISH instruments in conjunction with VENTANA detection kits and accessories. Tissue sections were deparaffinized and processed to unmask the antigenic sites. The CONFIRM anti-Ki-67 (30-9, Roche) Rabbit Monoclonal Primary Antibody was used. The sections were stained with OptiView DAB IHC Detection Kit in BenchMark instruments (Method GX, Roche).

### 2.7. AMH Immunohistochemistry

To detect AMH expression in Bouin-fixed paraffin-embedded from human biopsies and rat testis, we followed the protocol published by Rey et al. (1996) [10]. Briefly, testis sections were deparaffinized and hydrated by successive series of ethanol and then irradiated in a microwave oven (370 W for 5 min) in 0.01 mM sodium citrate buffer at pH 6.0, and then the endogenous peroxidase activity was blocked with 0.03% H_2_O_2_ in methanol absolute (Merck, Baltimore, MD, USA) for 30 min at RT. Nonspecific binding sites were blocked with BSA 10% and 20% avidin (Blocking kit, Vector Labs, Burlingame, CA, USA) in phosphate-buffered saline (PBS) for 30 min at RT. Sections were incubated with a rabbit anti-human recombinant AMH antibody (1:700, Tris-buffered saline containing 1% bovine serum albumin), generously gifted by Dr. Rodolfo Rey, overnight at 4 °C. Then sections were incubated with a goat biotinylated anti-rabbit secondary antibody (1/100, 1.5 mg/mL, Vector Labs) in PBS for 1 h at RT. The avidin–biotin system (Vectastain Elite ABC kit; Vector Laboratories, Burlingame, CA, USA) was applied for 45 min at RT and the reaction product was visualized by adding a diaminobenzidine substrate (Vector Laboratories). In the negative control, the first antibody was omitted. Sections were counterstained with hematoxylin, dehydrated by successive series of ethanol, and mounted.

### 2.8. Periodic Acid-Schiff (PAS) Staining

The Bouin-fixed paraffin-embedded biopsies and rat testes were deparaffinized and hydrated tissues were oxidized (0.5% periodic acid solution in distilled water) for 5 min and rinsed in distilled water. Tissues were then incubated in Schiff’s reagent for 15 min, washed in tap water for 5 min, and counterstained in Mayer’s hematoxylin for 1 min. Tissues were then washed in tap water for 5 min, rinsed in distilled water, dehydrated, and mounted.

### 2.9. Seminiferous Tubule Diameter and Wall Thickness Determination

The diameter of the STs in human biopsies and rat testis was determined using Image J software and calculated as the average between the diameter of the major and minor axes.

The seminiferous tubule wall thickness was studied in testis sections stained with PAS, determined using Image J software, and calculated as the mean value of the thicknesses of four regions of the STs.

### 2.10. Quantification of Sertoli Cells and Spermatogonia in Human Biopsies

The number of Sertoli cells and spermatogonia was determined by morphological criteria [11] from 6 nonconsecutive cross-sections per patient using a Nikon microscope at 40× magnification. Results were expressed as the number of Sertoli cells or the number of spermatogonia per ST.

**Figure 1:**
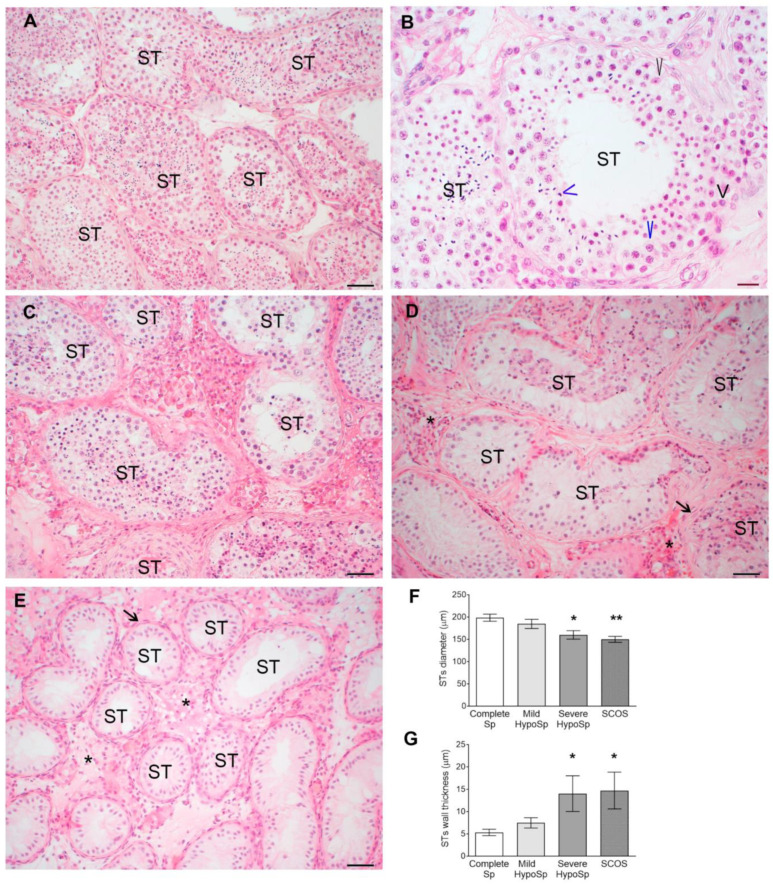
**Histopathology of testicular biopsies (A**–**E)**. Microphotographs of testicular biopsies stained with hematoxylin-eosin classified according to McLachlan et al.’s (2007) [13] criteria with modifications. Complete spermatogenesis (Complete Sp) (**A**,**B**): all cell populations of spermatogenesis were present in seminiferous tubules (STs), including spermatogonia (short black arrowhead), spermatocytes (long blue arrowhead), elongated spermatids (short blue arrowhead), and Sertoli cells (long black arrowhead). Mild hypospermatogenesis (Mild HypoSp) (**C**): the population of elongated spermatids was reduced in number in the STs. Severe hypospermatogenesis (Severe HypoSp) (**D**): a large reduction in the number of all germ cell populations was observed and no elongated spermatids were detected. Sertoli cell-only syndrome (SCOS) (**E**): STs showed total absence of mature germ cells and contained Sertoli cells. In (**D**,**E**), ST walls were thickened and fibrotic (black arrow) and increased cellularity was also observed in the interstitium (asterisk). Left panels, scale bar indicates 50 µm; right panels scale bar indicates 20 µm. Seminiferous tubule diameter (**F**) and thickness (**G**) were evaluated in 4–10 STs in four non-consecutive slices. Dunnett’s multiple comparisons test * *p* < 0.05 and ** *p* < 0.01 vs. STs diameter of Complete Sp. Dunn’s nonparametric test * *p* < 0.05 vs. STs thickness of Complete Sp.

Biopsies from patients with severe hypospermatogenesis (HypoSp) and SCOS have STs with smaller diameters than biopsies from the control group (Figure 1F). The number of counted Sertoli cells was corrected by a factor that considered this variable [12]. Germ cell loss reduces the length of STs by a proportional reduction in the diameter. The correction factor is the ST diameter from hypo spermatogenesis biopsies or the SCOS/STs diameter for the control group.

### 2.11. Determination of Sertoli Cell Number in Rat with Orchitis

In order to determine the number of Sertoli cells per testis, we ran a morphometric study using paraffin sections (equal section widths, 5 µm) following the method described by Mori and Christensen (1980) [14], with minor modifications. Briefly, sections were analyzed with a 40× objective, and the number of Sertoli cells per seminiferous tubule was counted. A total of 60 seminiferous tubules were analyzed per rat in each group (*n* = 4 rats per group).

The average nuclear diameter (D) of Sertoli cells was measured with a 100× Zeiss objective. The mean D of the nucleus was obtained using direct measurements of its long and short axes [14].

Numerical density (Na) was calculated by dividing the number of Sertoli cells in the seminiferous tubules by tubule area. The numerical density or number of Sertoli cells per unit of testicular volume (Nv) was then calculated using the Floderus equation [15]: Nv = Na/(D + T × 2h), where T is the section thickness, h is a correction factor to calculate nuclei diameters, and D is the average nuclear diameter calculated [14].

The total number of Sertoli cells per testis was then calculated by multiplying the final numerical density by the testis volume. The actual volume of each removed testis was estimated directly using a water displacement method [16].

### 2.12. Determination of Sperm Parameter in Rat Epididymis

Sperm was obtained from the epididymis cauda as previously described [17] and immediately incubated with human tubal fluid medium (HTF) containing 4 mg/mL of BSA at 37 °C for 10 min in a thermostatic bath [17]. To evaluate motility, sperm suspensions were placed on pre-warmed slides and analyzed subjectively under a light microscope (400×) [17]. Cells that moved in a forward direction were recorded as progressive motile sperm, and cells that vibrated or rotated in the same place were not considered. For sperm count, sperm suspensions were diluted in water to prevent sperm movement, and the number of sperm heads was recorded using a Neubauer Chamber under a light microscope (400×) [17]. Sperm viability was assessed by Eosin Y (Sigma, St. Louis, MO, USA, 0.5% *w*/*v*) exclusion, counting spermatozoa that did not incorporate the dye under a light microscope (400×) [17].

### 2.13. TUNEL Technique

Apoptosis was determined by the TUNEL assay in human biopsies and rat testis [18]. Testis sections were deparaffinized and hydrated by successive series of ethanol and then irradiated in a microwave oven (370W for 5 min) in 10 mM sodium citrate buffer at pH 6.0 and permeabilized with 0.1% Triton X-100 (Sigma-Aldrich) in 0.1% sodium citrate for 5 min at 4 °C. Non-specific labeling was prevented by incubating sections with blocking solution (2% blocking reagent, Roche Molecular Biochemicals GmbH, Mannheim, Germany) in 150 mM NaCl and 100 mM maleic acid at pH 7.5 for 30 min at RT. After 10 min incubation with terminal deoxynucleotidyl transferase (TdT) buffer (buffer TdT, 1×; CoCl_2_, 1×, Roche, Mannheim, Germany), apoptotic DNA was 3′-end labeled with digoxigenin-11-dideoxy-uridine triphosphate (4 μM Dig-ddUTP, Roche) by incubation with TdT (0.4 U/μL, Roche) in TdT buffer for 1 h at 37 °C. As an assay control, the TdT enzyme was replaced by the same volume of TdT buffer. Sections were then incubated with blocking solution (2% blocking reagent in 150 mM NaCl and 100 mM maleic acid, pH 7.5) for 30 min at RT, followed by the detection of Dig-dd-UTP with an alkaline phosphatase-conjugated anti-digoxigenin antibody (0.375 mU/μL, Roche) incubated for 2 h at RT. Sections were rinsed and equilibrated in alkaline phosphatase buffer (100 mM Tris–HCl, 100 mM NaCl, 50 mM MgSO_4_, pH 9.5) containing 1 mM levamisole (Sigma-Aldrich). Then, alkaline phosphatase substrates, nitroblue tetrazolium, and 5-bromo-4-chloro-3-indolyl-phosphate (NBT/BCIP, Roche) were added for 1 h. This reaction was stopped by washing with Tris–EDTA buffer (10 mM Tris–HCl, 1 mM EDTA, pH 8.0). Sections were rinsed in 95% ethanol for 24 h at RT, light counterstained with eosin, dehydrated by successive series of ethanol, and mounted. The number of TUNEL+ cells per ST was quantified in 3–6 nonconsecutive cross-sections per specimen representing normal spermatogenesis and severe HypoSp under a Nikon microscope at a magnification of 400×.

### 2.14. Statistical Analysis

Data were compared for statistical significance using GraphPad Prism version 8.0 (GraphPad Software Inc., La Jolla, CA, USA). Data are presented as the mean ± SEM. Differences were considered significant when *p* < 0.05.

## 3. Results

### 3.1. Histopathology of Testicular Biopsies from Infertile Patients and Hormonal Profile

Biopsies in the control group had complete spermatogenesis throughout the tissue and normal cellularity in the intertubular space (Figure 1A). We considered biopsies from patients with non-obstructive azoospermia with hypospermatogenesis in which all stages of spermatogenesis were present but reduced to a different degree; this classification also included areas with seminiferous tubules (STs) showing only Sertoli cells (Figure 1B,C). Given their histopathological heterogeneity, these biopsies were classified according to the number of elongated spermatids present in the STs as mild hypospermatogenesis when 5–20 spermatids were present (Figure 1B) and severe hypospermatogenesis when fewer than 5 elongated spermatids were present (Figure 1C).

In some patients (*n* = 6/28), biopsies were histopathologically heterogeneous, e.g., HypoSp combined with tubular hyaline atrophy (THA) to different degrees (patient 5: severe HypoSp (50%) and THA (50%); patient 15: complete Sp (80%) and THA (20%); patient 23: severe HypoSp (65%) and THA (35%); patient 28: severe HypoSp (70%) and THA (30%); patient 29: severe HypoSp (10%) and THA (90%); patient 36: mild HypoSp (70%) and THA (30%). The exceptions were biopsies diagnosed as SCOS.

In patients in which both testes were biopsied (*n* = 15), some biopsies (*n* = 6) presented different testicular histopathologies (patient 11: left testis: complete Sp, right testis ATH; patient 15: left testis: complete Sp and THA, right testis: complete Sp; patient 22: left testis: mild HypoSp, right testis: THA; patient 29: left testis: THA/severe HypoSp, right testis: complete Sp; patient 33: left testis: severe HypoSp, right testis: mild HypoSp; patient 36: left testis: mild HypoSp/THA right testis: HypoSp). The remainder were diagnosed as mild HypoSp (*n* = 3) and SCOS (*n* = 6).

The diameter of STs significantly decreased in testicular biopsies from patients with severe HypoSp and SCOS vs. Complete Sp, a consequence of spermatogenesis impairment and germ cell loss (Figure 1F).

STs wall thickness was greater in biopsies from patients with severe HypoSp and SCOS than in biopsies from complete Sp (Figure 1G and Figure S1: Biopsies, PAS staining).

Patients with severe HypoSp and SCOS had significantly higher FSH levels compared to patients with complete Sp (Table 1). Levels of LH, testosterone, and prolactin were similar in all groups studied (Table 1).

### 3.2. CD45+ Cells Increase in Testicular Biopsies

**Figure 2:**
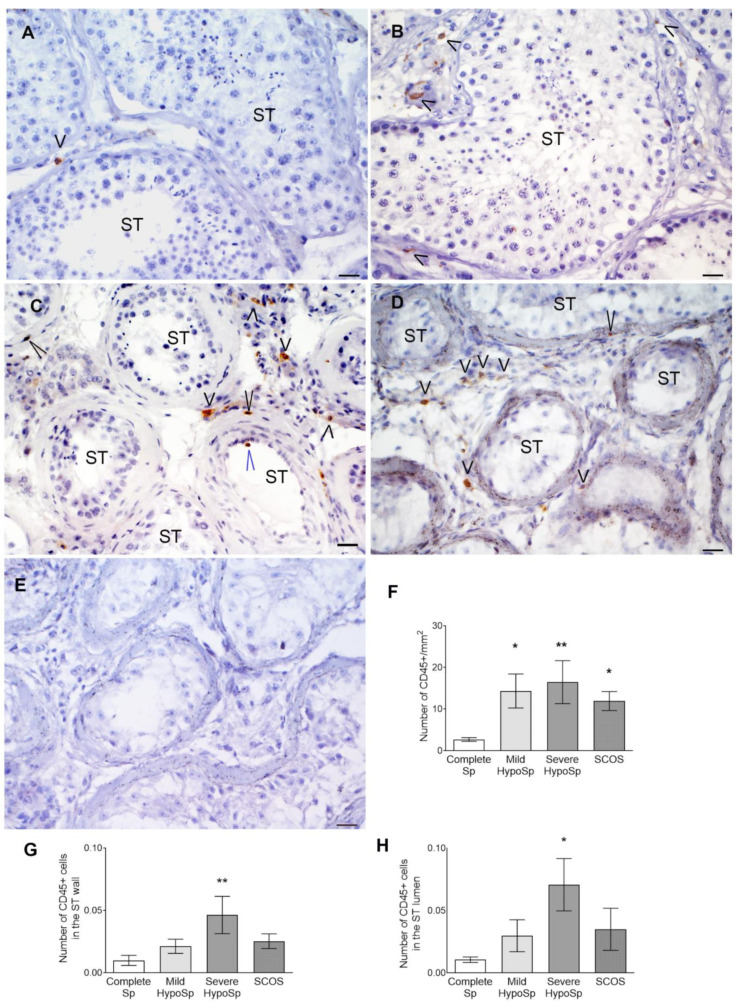
**Localization and quantification of immune cells (CD45+) in testicular biopsies.** Microphotographs of testicular biopsies showing the distribution of CD45+ cells. CD45+ in biopsies from patients with complete spermatogenesis (Complete Sp) (**A**), mild (Mild HypoSp) (**B**) and severe hypospermatogenesis (Severe HypoSp) (**C**), and Sertoli cell-only syndrome SCOS (**D**). In the negative control (**E**), the first antibody was omitted. Cells were localized in the interstitium (short black arrowhead), in the tubular wall (long black arrowhead), and in the lumen of the seminiferous tubules (STs) (long blue arrowhead). Scale bar indicates 20 µm. Total CD45+ cells were quantified (**F**) and analyzed according to their location in the STs: ST walls (**G**) and ST lumen (**H**) (six nonconsecutive slices per biopsy). Dunn’s nonparametric test * *p* < 0.05 and ** *p* < 0.01 vs. Complete Sp.

The total number of CD45+ cells/mm^2^ significantly increased in testicular biopsies from patients with mild and severe HypoSp and SCOS vs. patients with complete Sp (Figure 2A–E). Eighty-six percent of patients with mild, 80% with severe HypoSp, and 88% with SCOS showed more than twice the mean number of CD45+ cells in the biopsies vs. patients with complete Sp.

The majority of immune cells were present in the interstitium (Figure 2E–G). CD45+ cells present in the ST wall and inside the tubules significantly increased in patients with severe HypoSp vs. Complete Sp (Figure 2F,G).

### 3.3. Meiotic Germ Cells Are Reduced and Die by Apoptosis in Human Orchitis

**Figure 3:**
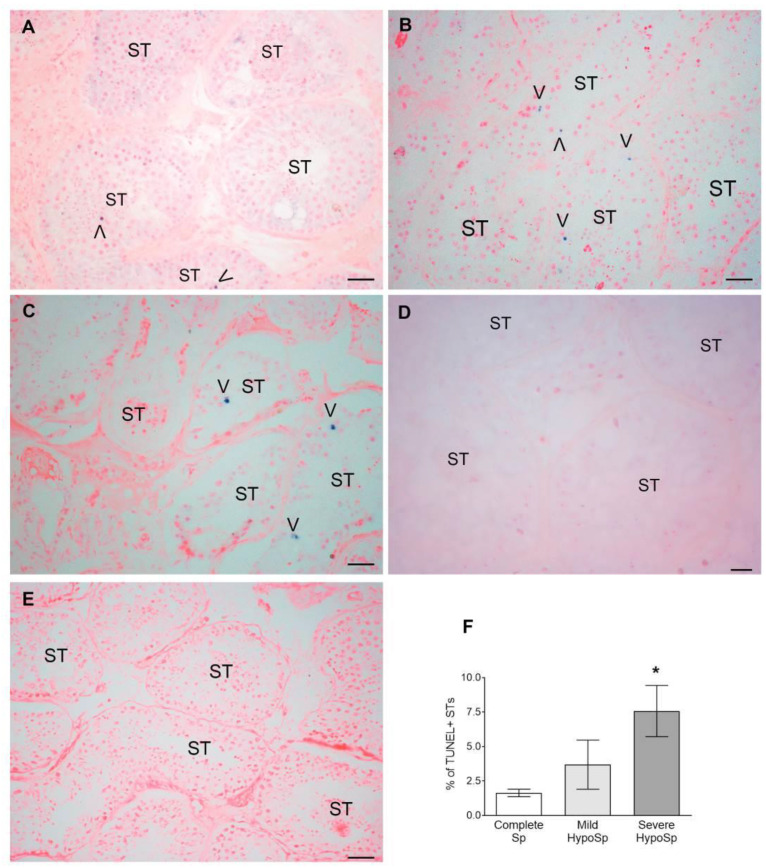
**Germ cell apoptosis.** Representative microphotographs showing TUNEL+ germ cells (arrowheads) in biopsies from patients with complete spermatogenesis (Complete Sp) (**A**), mild hypospermatogenesis (Mild HypoSp) (**B**), severe hypospermatogenesis (Severe HypoSp) (**C**), and Sertoli cell-only syndrome (SCOS) (**D**): notice that Sertoli cells were TUNEL-. In the negative control (**E**), the TdT enzyme was replaced by incubation buffer. Bar indicates 50 µm (**A**–**C**,**E**) and 20 µm Apoptosis was evaluated in six non-consecutive slices (**F**). Dunnett’s multiple comparisons test * *p* < 0.05 vs. Complete Sp.

The percentage of STs with apoptotic germ cells increases in biopsies from patients with severe HypoSp compared to the control group. Apoptotic germ cells are mainly localized in the adluminal region of the seminiferous tubules (Figure 3). Sertoli cells were TUNEL-negative in all patients studied (Figure 3).

### 3.4. The Number of Undifferentiated Type A and Differentiated Type B Spermatogonia Decreased in Human Orchitis

Undifferentiated type A pale and A dark spermatogonia were grouped to avoid biasing of morphological identification [19].

**Figure 4:**
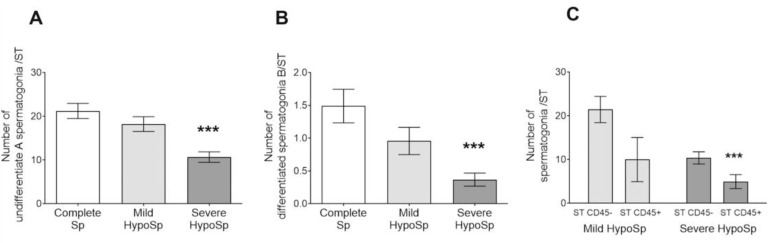
**Quantification of spermatogonia in testicular biopsies.** Undifferentiated A (dark and pale) (**A**) and differentiated B spermatogonia (**B**) were quantified in seminiferous tubule (ST) (six nonconsecutive slices per biopsy). Complete spermatogenesis (Complete Sp), mild hypospermatogenesis (Mild HypoSp), severe hypospermatogenesis (Severe HypoSp), and Sertoli cell-only syndrome (SCOS). Each column represents the mean ± SEM. Dunnett parametric test, *** *p* < 0.001 vs. Complete Sp. **Number of spermatogonia present in STs with or without immune cell infiltration in testicular biopsies** (**C**). Two-factor ANOVA followed by Bonferroni multiple comparisons test, *** *p* < 0.001 ST with CD45+ cell infiltrate (ST CD45+) vs. ST without cell infiltrate (ST CD45-) from patients with Severe HypoSp.

The number of undifferentiated and differentiated type B spermatogonia was reduced in biopsies of patients with severe HypoSp vs. Complete Sp (Figure 4A,B).

STs with CD45+ cells present in the wall and/or in the lumen showed fewer spermatogonia than those not invaded by immune cells (Figure 4C).

### 3.5. Analysis of Sertoli Cell Number and Ki67 Expression in Testicular Biopsies

**Figure 5:**
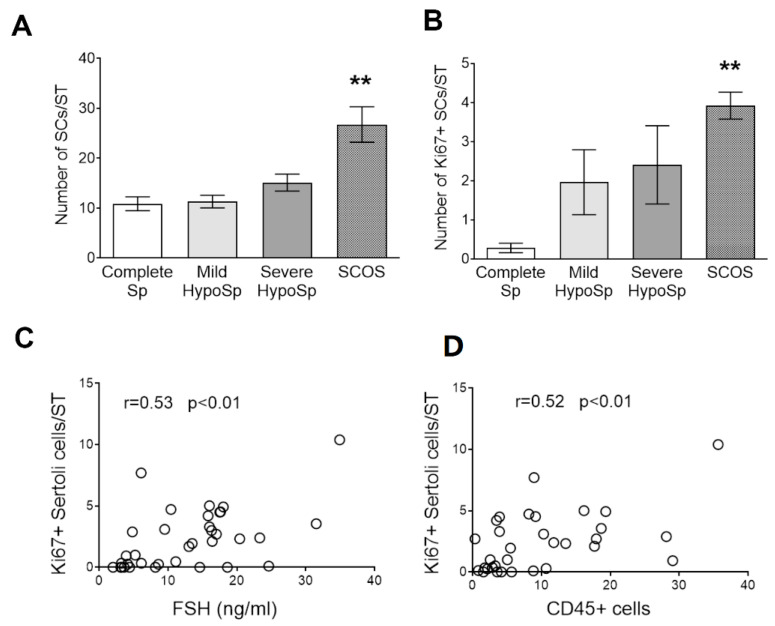
**Sertoli cells in testicular biopsies.** Number of Sertoli cells (**A**) and number of Ki67+ Sertoli (**B**) were quantified in 4–10 seminiferous tubules (STs) in six non-consecutive slices. Complete spermatogenesis (Complete Sp), mild hypospermatogenesis (Mild HypoSp), severe hypospermatogenesis (Severe HypoSp), and Sertoli cell-only syndrome (SCOS). Dunn’s nonparametric test ** *p* < 0.01 vs. Complete Sp. **Spearman correlation analysis between Ki67+ Sertoli cells and serum FSH** (**C**) **and between Ki67+ Sertoli and CD45+ cells** (**D**). Points represent values for each patient.

**Figure 6:**
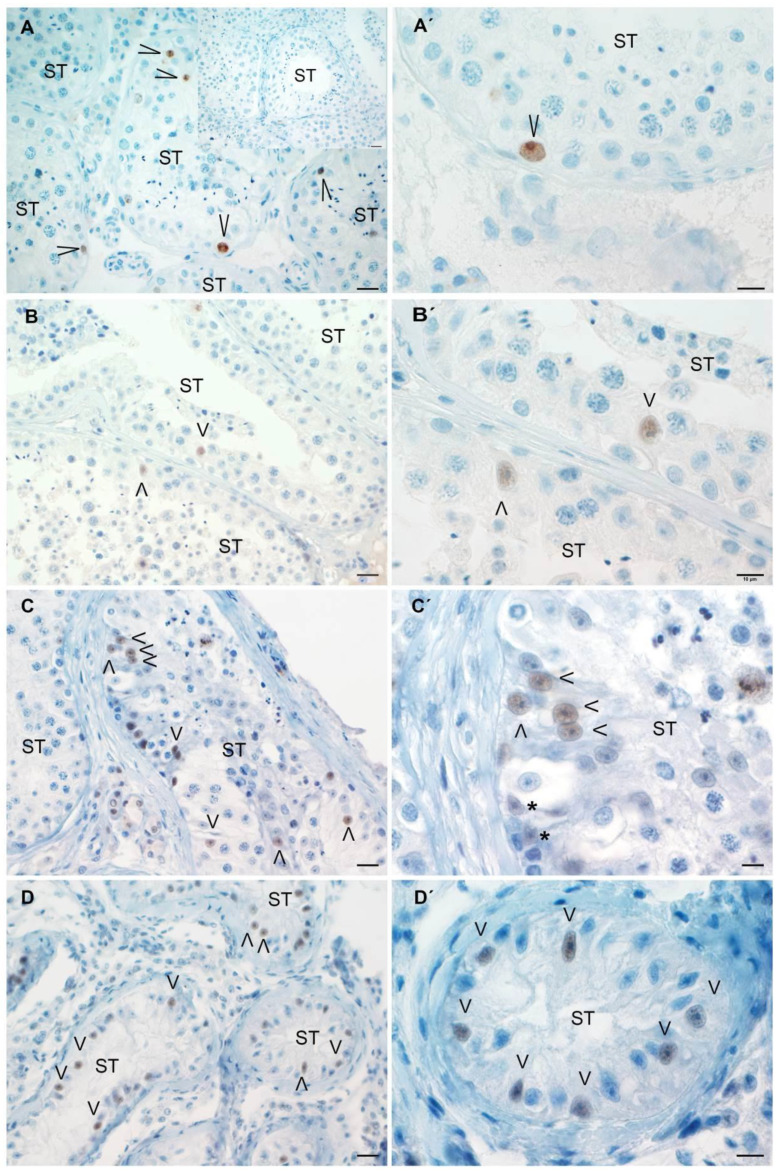
**Ki67 expression determined by immunoperoxidase.** Representative microphotographs: complete spermatogenesis (Complete Sp; **A**,**A′**), mild hypospermatogenesis (**B**,**B′**), severe hypospermatogenesis (**C**,**C′**), Sertoli cell-only syndrome (SCOS) (**D**,**D′**)**.** In the negative control, the first antibody was replaced by an incubation buffer (**Insert in A**). Sertoli cells (short arrowheads) express Ki67 in hypospermatogenesis and SCOS patients. Notice the immature phenotype of Sertoli cell nucleus (see the main document for better explanation) in (**B′**–**D′**). In (**B′**), an immature Sertoli cell nucleus with two nucleoli is shown (short arrowheads). In (**C′**), notice a group of Sertoli cells with immature phenotypes and others with mature phenotypes (asterisk), located in the basal zone of the same seminiferous tubule: with an indentation and nucleus with triangular shape. In patients with Complete Sp, Ki67+ germ cells are observed (long arrowheads), and Sertoli cells display mature phenotypes. Left panels, scale bar indicates 20 µm; right panels, bar indicates 10 µm.

The number of Sertoli cells per seminiferous tubule increased in patients with SCOS compared to the control group (Figure 5A). To determine the proliferative state of Sertoli cells, Ki67 expression was analyzed. Ki67+ Sertoli cells were observed in all groups of patients (Figure 6A–D). In severely damaged STs in mild HypoSp and severe HypoSp, and in SCOS, we observed that the Sertoli cell nucleus had immature morphological characteristics [20], was round to oval in shape, had regular contours and no indentations, had one or more nucleoli (not tripartite), and showed chromatin loosely distributed throughout the nuclear area (Figure 6B′–D′).

**Figure 7:**
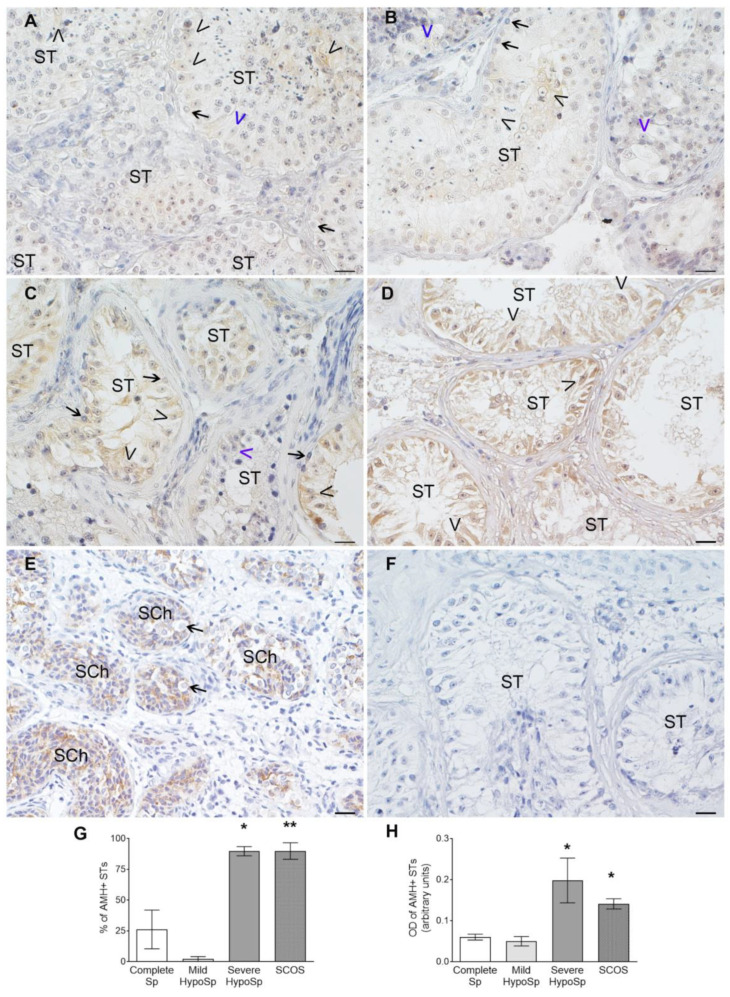
**AMH expression in Sertoli cells.** Representative microphotographs of testis sections processed for AMH detection using immunoperoxidase. In complete spermatogenesis (Complete Sp) (**A**) and mild hypospermatogenesis (Mild HypoSp), some Sertoli cells (SCs) are AMH+ (black arrowheads) whereas others are AMH- (blue arrowheads) (**B**). In severe HypoSp (Severe HypoSp) (**C**) and Sertoli cell-only syndrome (SCOS), most SCs are AMH+ (**D**)**.** Positive control: testicular biopsy of a prepubertal boy (7 months old), all SCs in the seminiferous chords (SCh) express AMH (**E**). Spermatogonia are AMH- (black arrow). In the negative control, the first antibody was replaced by an incubation buffer (**F**). Scale bar indicates 20 µm. **Percentage of seminiferous tubules with AMH+ Sertoli cells** (**G**). Each column represents the mean ± SEM. Dunn’s nonparametric test * *p* < 0.05 and ** *p* < 0.01 vs. Complete Sp. **Level of AMH expression in Sertoli cells** (**H**). Optical density (OD) was calculated from the analysis of microphotographs using the Image J program. Dunn’s nonparametric test * *p* < 0.05 vs. Complete Sp. Optical density in the negative control: mean ± SEM: 0.070 ± 0.005.

Patients with SCOS showed a significantly increased number of Ki67+ SCs/ST (Figure 7A). Sertoli cell proliferation is controlled by a plethora of factors that stimulate or suppress cell division [21]. FSH and cytokines (TNFα, IL1β, and IL6) are known to promote Sertoli proliferation. The number of Ki67+ Sertoli cells correlates positively with the levels of serum FSH (Figure 7B) and the presence of CD45+ cells (Figure 7C).

### 3.6. Analysis of AMH Expression in Testicular Biopsies with Orchitis

AMH expression in Sertoli cells was observed in patients with complete Sp (*n* = 3/9) and mild HypoSp (*n* = 1/9), AMH+ Sertoli cells were scattered throughout the STs (Figure 7A,B). In patients with severe HypoSp and SCOS, most of the Sertoli cells in the STs expressed AMH (Figure 7C,D) and the percentage of AMH+ STs increased significantly vs. those with complete Sp (Figure 7G). In patients with severe HypoSp and SCOS, the level of AMH expression in Sertoli cells was highestand it was lowest in patients with complete and mild HypoSp (Figure 7H).

### 3.7. EAO Histopathology and Hormonal Profile

**Figure 8:**
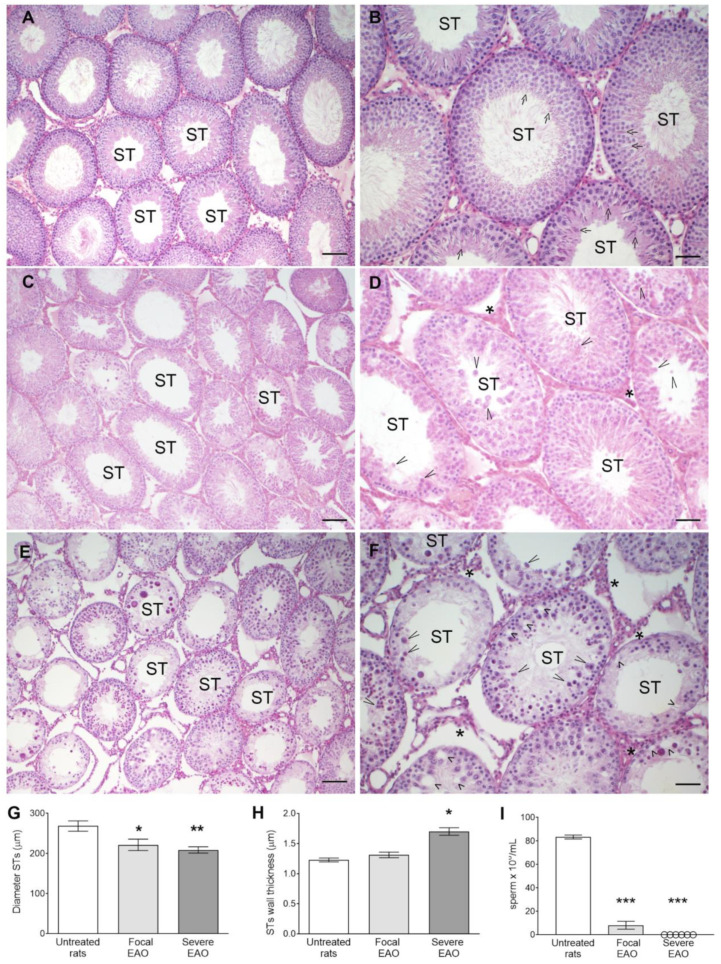
**Histopathology, seminiferous tubule diameter (G), wall thickness (H), and sperm (I) count in EAO.** Representative micrographs of testis sections of untreated rats (**A**,**B**) showing complete spermatogenesis: all seminiferous tubules (STs) and the interstitial compartment were preserved; in focal orchitis (**C**,**D**), elongated spermatids (arrow) were lost in most STs, degenerated round spermatids and spermatocytes (long arrowhead) were present in the lumen of some STs, vacuolization of Sertoli cells occurred (short arrowhead), and increased cellularity was present in the interstitial compartment (asterisk); in severe orchitis (**E**,**F**), STs lost most of the spermatid and spermatocyte populations. In most STs, Sertoli cells and spermatogonia were the main cells present; cellularity was increased in the interstitium. In (**G**,**H**): 60 STs were analyzed in testis sections from untreated (*n* = 7); Focal EAO, (*n* = 7) and Severe EAO (*n* = 8) rats. Dunn’s multiple comparisons test * *p* < 0.05 and ** *p* < 0.01 vs. untreated rats. In (**I**): sperm was evaluated in the epididymis cauda from untreated (*n* = 11); Focal EAO, (*n* = 9) and Severe EAO (*n* = 6) rats. Dunn’s multiple comparisons test *** *p* < 0.001 vs. untreated rats.

The main histological feature of focal orchitis is the sloughing and death by apoptosis of meiotic germ cells, spermatids, and spermatocytes. These cells are often observed in the lumen of the STs. However, STs with preserved spermatogenesis are often found (Figure 8C,D).

There is a progressive loss of meiotic germ cells, so that in the severe phase, the only cells present in most STs are spermatogonia and Sertoli cells (Figure 8E,F).

In EAO, the percentage of STs with apoptotic germ cells is significantly increased compared to untreated rats (mean ± SEM, Untreated rats (*n* = 5): 20.44 ± 1.925; focal OAE (*n* = 5): 37.40 ± 5.136 *; severe OAE (*n* = 4): 42.92 ± 9.399 *, Dunnett’s multiple comparisons test, * *p* < 0.05 vs. untreated rats). As we reported previously, TUNEL+ germ cells in the adluminal compartment of STs are more numerous than those located in the basal compartment [7,18].

The seminiferous tubule diameter significantly decreased in rats with severe EAO vs. untreated rats (Figure 8H). In cases of atrophic seminiferous tubules with severe sloughing of the germinal epithelium, the tubular wall usually undergoes fibrosis, visualized as thickening due to the accumulation of an extracellular matrix. Thickening of the seminiferous tubule wall was observed in severe EAO (Figure 8I and Figure S2: EAO, PAS staining).

In the testicular interstitium, cellularity increased due to infiltration of immune cells [6] and hyperplasia of Leydig cells [8]. These events occur concomitantly with the activation of angiogenesis [6] (Figure 8B,D).

During inflammatory spermatogenesis, sperm production is progressively reduced, resulting in a significant decrease in sperm count in focal and severe EAO rats compared to untreated rats (Figure 8I), as well as a decrease in sperm viability and motility, (% of sperm viability, mean ± SEM, Untreated rats (*n* = 5): 78.00 ± 1.91; focal OAE (*n* = 5): 17.52 ± 5.41 **; severe OAE (*n* = 5): 0.0 ± 0.0 **, Dunn’s multiple comparisons test, ** *p* < 0.01 vs. untreated rats) (% of sperm motility, mean ± SEM, Untreated rats (*n* = 5): 83.41 ± 1.68; focal OAE (*n* = 5): 8.14 ± 3.37 **; severe OAE (*n* = 5): 0.0 ± 0.0 **, Dunn’s multiple comparisons test, ** *p* < 0.01 vs. untreated rats).

In EAO, prolactin levels were similar to untreated rats (ng/mL, mean ± SEM, Untreated rats (*n* = 11): 13.10 ± 1.99; focal OAE (*n* = 11): 14.93 ± 1.80; severe OAE (*n* = 11): 9.37 ± 2.78 and Table S1: Serum hormonal levels in human and rat orchitis). As we reported, LH is similar in untreated rats (Table S1); testosterone levels were variable in focal EAO (equal, increased, or decreased compared to untreated rats) [3,8,9], whereas in severe EAO, they were higher vs. untreated rats [8,15] (Supplemental Table S1); FSH levels were significantly increased in focal and severe EAO vs. untreated rats (Supplemental Table S1). 

### 3.8. Study of Sertoli Cells in Rats with EAO

**Figure 9:**
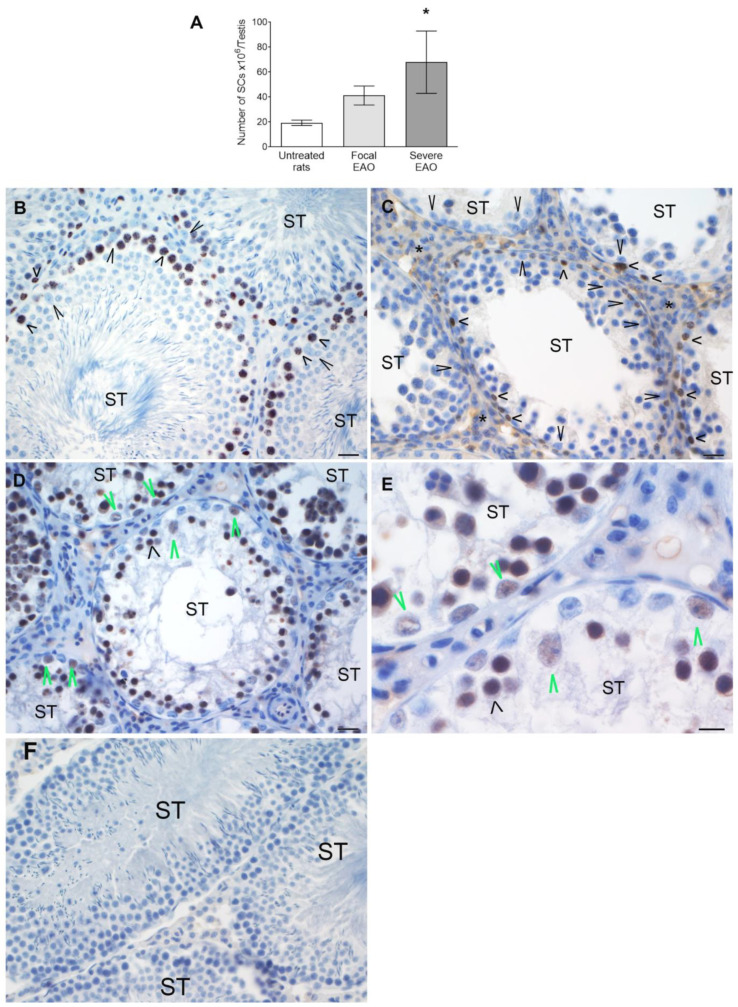
**Number of Sertoli cells and Ki67+ expression in Sertoli cells in rats with EAO.** Sertoli cells were quantified in 100 seminiferous tubules (STs). (**A**) Untreated rat *n* = 4; Focal EAO, *n* = 4; Severe EAO, *n* = 4. Dunn’s multiple comparisons test * *p* < 0.05 vs. untreated rat. Ki67 was determined using immunoperoxidase, representative microphotographs of testis sections of untreated (**B**), focal EAO (**C**), and severe EAO rats (**D**,**E**). In untreated and focal EAO testis, Sertoli cells (large arrowheads) did not express Ki67, unlike premeiotic germ cells (spermatogonia and preleptotene spermatocytes) (short arrowheads). Many cells in the interstitial compartment (*) are Ki67+. In severe EAO, Sertoli cells expressed Ki67 (large green arrowheads), and some also had immature phenotypes (hashtag). In the negative control (**F**), the first antibody was replaced by incubation buffer. Scale bar indicates 20 µm and (10 µm (**E**)).

The number of Sertoli cells per testis increased significantly in severe orchitis vs. untreated rats (Figure 9A). We observed Ki67+ Sertoli cells only in the testes of rats with severe EAO. The cells were located in severely damaged STs where spermatogonia were the only germ cells present (Figure 9D). Regarding their nuclear characteristics, Sertoli cells with mature and immature phenotypes were located in the same ST (Figure 9E). Sertoli cells in untreated rats with focal EAO were Ki67- and displayed a mature phenotype (Figure 9B,C).

**Figure 10:**
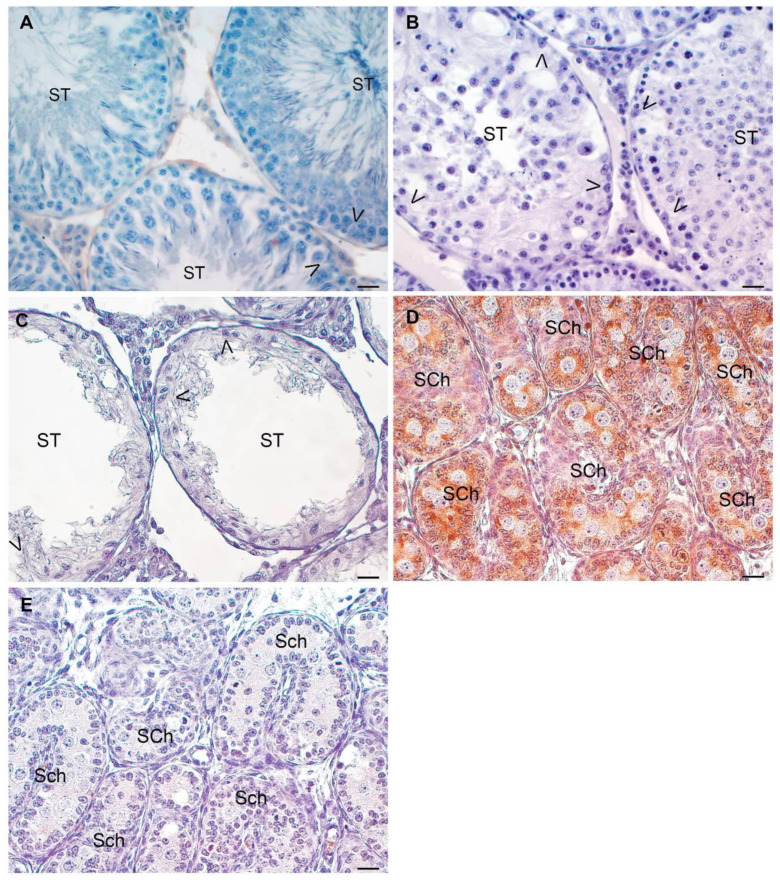
**AMH expression in rat Sertoli cells.** Representative microphotographs of testis sections of untreated (**A**), focal EAO (**B**), and severe EAO (**C**) rats processed for AMH detection by immunoperoxidase. Sertoli cells (arrowheads) in all groups studied did not express AMH. Positive control: prepubertal rat testis (2 d) (**D**) showing AMH expression in Sertoli cell cytoplasm. The first antibody was replaced by incubation buffer in the negative control (**E**). Scale bar indicates 20 µm.

As expected, neither Sertoli cells from untreated adult rat testes nor Sertoli cells from EAO rat testis express AMH (Figure 10).

### 3.9. Human Orchitis and Experimental Autoimmune Orchitis (EAO)

A summary of the main histopathological findings in biopsies from patients with orchitis and in rats with EAO is presented in Table 2.

In infertile patients with HypoSp, we observed two different degrees of spermatogenesis alteration, mild and severe; patients with severe HypoSp had more frequently thickened and hyalinized seminiferous tubules, a lower number of spermatogonia, and a higher number of immature Sertoli cells (Table 2). Similar levels of testicular damage and seminiferous tubule and Sertoli cell changes were observed in focal and severe EAO (Table 2).

## 4. Discussion

In this work, we studied human testicular biopsies from infertile patients with alterations in spermatogenesis and azoospermia and their association with idiopathic inflammatory conditions. Although the histopathology of spermatogenesis in infertile patients is well known, the overall relationship between germ cell and Sertoli cell alterations and local inflammatory cells in humans has not been evaluated.

Our group has been studying, for many years, how inflammatory mediators produced by immune cells infiltrating the testis alter testicular physiology in the rat model of EAO [7,22]. The main findings in the present study in testis biopsies from patients with HypoSp or SCOS were compared with those from the EAO model to consider whether testicular pathology in patients with idiopathic orchitis can be explained by the mechanisms involved in rat orchitis spermatogenesis impairment.

Classification criteria of testicular biopsies based on the number of spermatids per seminiferous tubule (ST) defined three groups reflecting progressive histopathological damage from mild HypoSp to severe HypoSp, although we found mixed histopathological phenotypes. The exceptions were biopsies diagnosed as SCOS. In some patients, left and right testes revealed different degrees of spermatogenesis impairment: all had complete or mild HypSp in one testis and a more severe phenotype in the contra lateral testis.

We determined the prevalence of CD45+ immune infiltrates in testicular biopsies; we observed that these cells are present in 88% and 78% of biopsies from patients with HypoSp and SCOS, respectively, at least twice the number of CD45+ cells as those found in biopsies from patients with Complete Sp. Pilatz et al. (2019) [2] observed that testicular leukocyte infiltration in the testes of NOA infertile patients was unrelated to the presence of pathogens and signs of inflammation within the urogenital tract.

Lymphocytes T (CD8+ and CD4+), macrophages (CD68+ and CD163+), and mast cells appear to be the immune cells most represented in the CD45+ cell population observed in testicular biopsies [23,24,25,26]. These cells express proinflammatory cytokines such as IL6, TNFα, IFNγ, IL22, IL17, and nitric oxide synthase [23], factors with a proven effect on testicular dysfunction in EAO [6].

Undifferentiated spermatogonia type A and differentiated spermatogonia type B populations decreased in patients with HypoSp, which negatively correlates with infiltrating immune cells. Moreover, STs with immune cell infiltrates, either in the lumen or in the ST wall, showed a more severe decrease in the number of spermatogonia than STs with no immune infiltrates. These results allow us to infer an association between quantitative alterations of spermatogenesis and the local inflammatory microenvironment generated by the presence of CD45+ cells.

The quantification of spermatogonia in testicular biopsies from azoospermic patients has been reported. Hentrich et al. (2011) [27] showed a decrease in the number of undifferentiated type A spermatogonia (identified by immunofluorescence with anti-UTF-1 antibody). This study did not evaluate the severity of the pathology or the presence of cell infiltrates.

Several mechanisms, mediated by cell-released inflammatory agents, might explain the decrease in spermatogonia population in infertile patients. In relation to apoptosis, the death of spermatogonia might not be the major phenomenon accounting for the reduction in this population since in patients with mild and severe HypoSp, we observed that apoptotic germ cells were located mostly in the luminal region of the STs, where only spermatids and spermatocytes are found. However, we cannot exclude that these cells are spermatogonia or preleptotene spermatocytes that undergo apoptosis as a consequenceof the loss of adhesion from the ST basement membrane. Moreover, the TUNEL technique identifies apoptotic cells at late stages of the death process, and therefore these cells could be observed only when they reached the adluminal region of the STs. Lin et al. (1997) [28] reported an increase in the number of apoptotic germ cells in the adluminal compartment of STs in testicular biopsies from patients with non-obstructive azoospermia in concordance with the results presented here.

In focal and severe EAO, the number of TUNEL+ spermatogonia and preleptotene spermatocytes (basal germ cells of the STs) is similar to untreated rats, whereas the number of adluminal-located round spermatids and spermatocytes that are TUNEL+ significantly increased [7]. TNFα and IL6 induce apoptosis in IL6R+ and TNFR1+ mature germ cells together with nitric oxide (NO) [9,18]. NO released by testicular macrophages arrests the cell cycle of undifferentiated spermatogonia (CD9+). The cell cycle arrest of CD9+ spermatogonia leads to a reduction in the number of undifferentiated and differentiated (c-Kit+) spermatogonia [7], limiting the clonal expansion of these cells, as we verified by the reduced number of spermatogonia in S phase. Reduced proliferation of spermatogonia also occurs in infertile patients with NOA [29].

Before puberty, Sertoli cells in the normal testis express a high level of AMH driven by specific transcription factors (SOX9, SF1, WT1, and GATA4), FSH and estrogens. During puberty, when Sertoli cells express androgen receptors (AR), intratesticular levels of testosterone override AMH stimulation by estrogens and FSH, and the hormone decreases. The direct effects of sex steroids on AMH transcription are mediated by AR and estrogen receptor α action on AMH promoter sequences [30]. A study by Lan et al. (2013) [31] showed that in a group of SCOS patients, the upregulation of SOX9 in conjunction with the downregulation of AR in Sertoli cells may explain the upregulation of AMH expression in these cells We reported here that not only patients with SCOS but also those with severe HypoSp highly expressed AMH in 67% and 100% of patients, respectively, having high FSH levels.

It has long been demonstrated that only immature Sertoli cells proliferate and that, at puberty, when Sertoli cells mature, their proliferation ceases and they form the blood–testis barrier (BTB) and maintain the developing germ cells. During Sertoli cell maturation, testosterone, thyroid hormones (T3 and T4), and retinoic acid induce the expression of differentiation markers in Sertoli cells and, simultaneously, the activation of cell cycle inhibitory proteins p21Cip1 and p27Kip1 [32].

Ki67 expression in Sertoli cells in patients with mild and severe HypoSp and SCOS, even in those with complete Sp, indicates that these cells are not quiescent. Moreover, nuclei of Sertoli cells are histopathologically different from completely resting Sertoli cells present in the healthy adult human testis. In patients with SCOS, the number of Sertoli cells and those that are Ki67+ is increased, indicating their current proliferative state. FSH is the main mitogenic factor that regulates the proliferation of immature Sertoli cells [21]. High levels of FSH and an increased number of immune cells correlate to an increased number of Sertoli cells in human orchitis. We speculate that sustained high FSH levels, together with proinflammatory cytokines, support the mitotic activity of these cells.

Zhao et al. (2020) [33] showed that Sertoli cells from idiopathic non-obstructive azoospermic patients (iNOA) are physiologically immature, express higher levels of mitotic genes, and proliferate faster than normal adult Sertoli cells in vitro. Furthermore, immature Sertoli cells do not support germ cell survival as efficiently as adult cells.

Sertoli cells in adult rat testis with severe inflammation (severe EAO) outnumber those present in the untreated normal testis, and Sertoli cells in very damaged STs are Ki67+.

Sertoli cells are capable of proliferation into adulthood in the transition region between the STs and the rete testis in Wistar rats [34]. These undifferentiated Sertoli cells may compose a subpopulation of Sertoli cell progenitors that reside in a specific microenvironment capable of growing the STs length under physiological or even experimental conditions if needed from this particular testis region [35]. In fact, some of the BrdU-positive Sertoli cells were able to move from the transition region into the STs core region, thereby contributing to the spermatogenic Sertoli cell population [36].

The transition zone is morphologically similar in laboratory rodents (rats, mice, and hamsters) and humans and is populated by an increased number of immune cells [35]. As for immature rat Sertoli cell proliferation, it has been shown that IL1α, IL1β, and TNFα increase DNA synthesis and Sertoli cell number in vitro [37,38]. Coincidentally, in EAO in mice, the tubuli recti is the first site to show immune cell infiltrates, which then spread throughout the testis [4]. Considering that IL1α and TNFα are highly produced in orchitis [4,6,24,25], the possible role of these cytokines in Sertoli cell proliferation under inflammatory conditions is feasible.

We speculate that in the pathologically inflamed testis, immature KI67+ Sertoli cells may arise from the transition zone to enhance spermatogenesis from remnant spermatogonia.

Serum testosterone levels are within the normal range in patients with severe HypoSp and SCOS. We did not measure intratesticular testosterone levels, and consequently, we cannot rule out this factor as being involved in testicular failure. Lardone et al. (2013) [39] showed that intratesticular testosterone concentrations were higher in tissues with complete or focal SCOS and mixed atrophy than in patients with normal spermatogenesis and validated that the severity of spermatogenic impairment is associated with major morphological and functional disturbance of the Leydig cell compartment. In concordance, patients in this study with SCOS (*n* = 2) and severe HypoSp (*n* = 2) also displayed Leydig cell hyperplasia. In EAO, hypertrophy and hyperplasia of Leydig cells are associated with increased levels of intratesticular testosterone [8,9,15]. Macrophages and NO released by these cells act as positive local modulators of testosterone secretion [9] in EAO.

At puberty, terminal differentiation of Sertoli cells involves the formation of inter-Sertoli cell tight junctions and the establishment of the BTB [21]. As spermatogenesis progresses, meiotic germ cells appear in the STs, expressing new antigens for the immune system. STs with dysfunction and or impairment of tight junctions constitute a leakage site for the exit of those antigens normally sequestered in the adluminal compartment by BTB. A loss of integrity in the design of BTB associated with the disorganization or dysfunction of tight junction proteins (ZO-1, occludin and claudin-11) has been associated with the disruption of spermatogenesis in NOA patients in whom testicular sperm retrieval was not possible [40].

In EAO, the release of sequestered meiotic germ cell antigen sustains the chronic inflammatory status of the testis in concert with the testicular draining lymph node [41,42]. Breakdown of the BTB caused by IL6, IL17, TNFα, and NO associated with a decrease in occludin expression and de-localization of claudin-11 and ZO-1 increase BTB permeability [6,43]. These proinflammatory factors present in the inflamed testis of infertile patients can alter the structure and function of tight junctions between Sertoli cells, contributing to the inflammatory process [23,44].

The preservation of undifferentiated type A spermatogonia in the testicular biopsies of infertile patients suggests that spermatogenesis might be improved by controlling inflammation and Sertoli cell nursing-scaffold function through pharmacological treatment and/or Sertoli cell/Sertoli cell-derived exosomes therapy [45,46,47]. The heterogeneity of testicular histopathology observed in these patients may be a positive factor in favor of treatment success.

This work supports EAO as a valuable model for studying the impact of inflammatory processes on testicular function and also as a useful tool for the implementation of specific therapies that can provide a solution for the treatment of testicular pathologies associated with an inflammatory condition.

## 5. Conclusions

We demonstrated a high prevalence of orchitis in testicular biopsies of azoospermic patients with HypoSp and SCOS. We showed that in testicular biopsies from infertile patients, as found in severe orchitis in rats, FSH levels and immune cell infiltration and deep changes in the phenotype and behavior of Sertoli cells are associated with a reduction in spermatogonial populations, leading to azoospermia. Also, Sertoli cells demonstrated responsiveness to changes in the testicular microenvironment.

Based on the common hormonal profile, germ cell, and Sertoli cell behavior shared by orchitis in rats and humans, we support EAO as a useful model for studying and implementing therapies to provide a solution for the treatment of testicular pathologies associated with inflammation and spermatogenesis dysfunction.

## Figures and Tables

**Table 1:** Serum hormonal levels in patients.

	Testosterone	LH	FSH	PRL
	(RV: 1.3–8.2 ng/mL)	(RV: 0.8–8.6 ng/mL)	(RV: 0.95–11.95 ng/mL)	(RV: 2.5–17.0 ng/mL)
Complete Sp	4.24 ± 0.71 (*n* = 8)	4.1 ± 0.57 (*n* = 8)	5.21 ± 1.06 (*n* = 8)	8.9 ± 1.16 (*n* = 9)
Mild HypoSp	4.77 ± 0.86 (*n* = 8)	4.1 ± 0.57 (*n* = 9)	9.55 ± 2.57 (*n* = 9)	11.29 ± 2.44 (*n* = 9)
Severe HypoSp	3.95 ± 0.46 (*n* = 8)	9.5 ± 2.1 (*n* = 10)	21.4 ± 4.1 (*n* = 10) *	14.03 ± 2.67 (*n* = 10)
SCOS	5.01 ± 0.68 (*n* = 8)	6.28 ± 0.79 (*n* = 9)	20.01 ± 1.96 (*n* = 10) *	11.54 ± 2.31 (*n* = 6)

Complete spermatogenesis (Complete Sp), mild hypospermatogenesis (Mild HypoSp), severe hypospermatogenesis (Severe HypoSp), and Sertoli cell-only syndrome (SCOS). RV: reference value. Values represent mean ± SEM. Dunn’s nonparametric test * *p* < 0.05 vs. Complete Sp.

**Table 2:** Pathological events in human and rat orchitis.

	Human Mild HypoSp	Human Severe HypSp	SCOS	Rat Focal EAO	Rat Severe EAO
Sperm count	azoospermia	azoospermia	azoospermia	decreased	azoospermia
Immune cell infiltrates	increased	increased	increased	increased [6]	increased [6]
STs diameter	unchanged	reduced	reduced	reduced	reduced
STs thickness	unchanged	increased	increased	unchanged	increased
Undifferentiated SPG	unchanged	reduced	nd	reduced [7]	reduced [7]
Differentiated SPG	unchanged	reduced	nd	reduced [7]	reduced [7]
Sertoli cell number	unchanged	unchanged	increased	unchanged	increased
Ki67+ Sertoli cells	unchanged	unchanged	increased	-	+
AMH+ Sertoli cells	-	increased	increased	-	-

Data from patients with mild hypospermatogenesis (Mild HypoSp), severe hypospermatogenesis (Severe HypoSp), and Sertoli cell-only syndrome (SCOS) were compared with those with Complete spermatogenesis. Data from focal and severe experimental autoimmune orchitis (EAO) were compared with those from untreated rats. No expression: -. Not determined: nd.

## Data Availability

Data is unavailable. National Law of Protection of Personal Data N° 25.326 on Confidentiality and Dissociation of Data, in force in our country (URL: https://www.argentina.gob.ar/normativa/nacional/ley-25326-64790, accessed on 1 March 2024) states that medical records and the results obtained during the research will be kept confidential.

## Supplementary Materials

The following supporting information can be downloaded at: https://www.mdpi.com/article/10.3390/biology13040278/s1. Table S1. Serum hormonal levels in human and rat orchitis; Figure S1 PAS staining of human biopsies; Figure S2 PAS staining of rat testis sections.
