# Supplementary material for: Impaired Spermatogenesis in Infertile Patients with Orchitis and Experimental Autoimmune Orchitis in Rats
Source: Biology (Basel). 2024 Apr 19;13(4):278. doi: 10.3390/biology13040278 (PMC11048156; doi:10.3390/biology13040278)
Supplement: Supplementary file 1 [file biology-13-00278-s001.zip › Figure S1 Biopsies PAS staining.pdf]

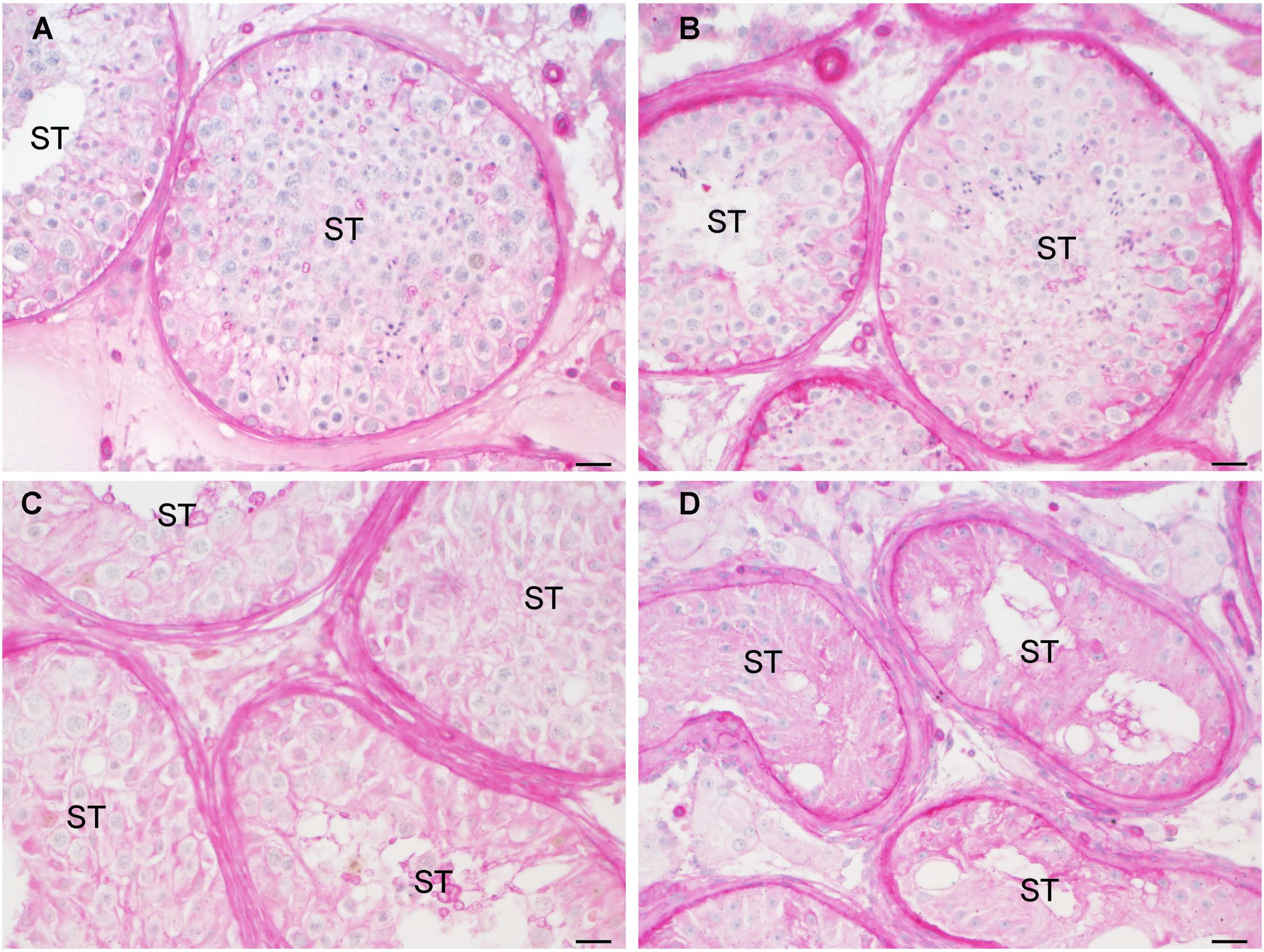

Figure S1: Biopies from patients with complete spermatogenesis (A), mild (B), severe hypospermatogenesis and SCOS (D) were stained with PAS.
Note seminiferpus tubule (ST) thickness in C and D. Scale bar indicates: 20 µm.
