# Supplementary material for: Impaired Spermatogenesis in Infertile Patients with Orchitis and Experimental Autoimmune Orchitis in Rats
Source: Biology (Basel). 2024 Apr 19;13(4):278. doi: 10.3390/biology13040278 (PMC11048156; doi:10.3390/biology13040278)
Supplement: Supplementary file 1 [file biology-13-00278-s001.zip › revision Table S1. Serum hormonal levels in human and rat orchitis.pdf]

Table S1. *Serum hormonal levels in human and rat orchitis*

	Human<br>Mild HypoSp	Human<br>Severe HypoSp	SCOS	Rat<br>Focal EAO	Rat<br>Severe EAO
**Serum Testosterone**	unchanged	unchanged	unchanged	unchanged [9] /decreased [3,8]	increased [15]
**Serum LH**	unchanged	unchanged	unchanged	unchanged [8,15]	unchanged [15]
**Serum FSH**	unchanged	increased	increased	increased [8,15]	increased [15]
**Serum PRL**	unchanged	unchanged	unchanged	unchanged	unchanged

Data from patients with Mild hypospermatogenesis (Mild HypoSp), severe hypospermatogenesis (Severe HypoSp) and Sertoli cell-only syndrome (SCOS) were compared with the data from patients with Complete spermatogenesis.

Data from rats with focal and severe orchitis were compared with the non-immunized rats.

PRL: prolactin.
